# Required and Voluntary Occupational Use of Hazard Controls for COVID-19 Prevention in Non–Health Care Workplaces — United States, June 2020

**DOI:** 10.15585/mmwr.mm7007a5

**Published:** 2021-02-19

**Authors:** Rachael M. Billock, Matthew R. Groenewold, Hannah Free, Marie Haring Sweeney, Sara E. Luckhaupt

**Affiliations:** 1 Division of Field Studies and Engineering, National Institute for Occupational Safety and Health, CDC.

Certain hazard controls, including physical barriers, cloth face masks, and other personal protective equipment (PPE), are recommended to reduce coronavirus 2019 (COVID-19) transmission in the workplace (*1*). Evaluation of occupational hazard control use for COVID-19 prevention can identify inadequately protected workers and opportunities to improve use. CDC’s National Institute for Occupational Safety and Health used data from the June 2020 SummerStyles survey to characterize required and voluntary use of COVID-19–related occupational hazard controls among U.S. non–health care workers. A survey-weighted regression model was used to estimate the association between employer provision of hazard controls and voluntary use, and stratum-specific adjusted risk differences (aRDs) among workers reporting household incomes <250% and ≥250% of national poverty thresholds were estimated to assess effect modification by income. Approximately one half (45.6%; 95% confidence interval [CI] = 41.0%–50.3%) of non–health care workers reported use of hazard controls in the workplace, 55.5% (95% CI = 48.8%–62.2%) of whom reported employer requirements to use them. After adjustment for occupational group and proximity to others at work, voluntary use was approximately double, or 22.3 absolute percentage points higher, among workers who were provided hazard controls than among those who were not. This effect was more apparent among lower-income (aRD = 31.0%) than among higher-income workers (aRD = 16.3%). Employers can help protect workers from COVID-19 by requiring and encouraging use of occupational hazard controls and providing hazard controls to employees (*1*).

Although many workplaces have implemented CDC (*1*) and Occupational Safety and Health Administration (OSHA) (*2*) guidance on engineering and administrative controls to prevent COVID-19, certain occupations might necessitate close contact among workers. Widespread occupational use of masks as source control or of physical barriers, masks, or other PPE to minimize exposure is likely to reduce COVID-19 transmission among workers and their communities. Workers with lower incomes have higher prevalences of comorbidities that increase risk for severe COVID-19–associated illness (*3*) and might face barriers to voluntary occupational hazard control use, including difficulty accessing masks or other PPE and reduced ability to independently choose to use hazard controls (*4*).

Survey questions were administered by Porter Novelli Public Services through the SummerStyles survey, one in a series of annual surveys. Respondents were recruited randomly by mail using address-based probability sampling to represent the noninstitutionalized, adult U.S. population; surveys were conducted via an online panel in English, and data were weighted to match U.S. Current Population Survey (*5*) proportions.* The June 2020 survey had a response rate of 62.7% (4,053) and included questions on COVID-19–related workplace characteristics. Respondents who did not work (1,409; 35%) or primarily worked from home after March 1, 2020 (819; 20%), used PPE at work before the COVID-19 pandemic (1,038; 26%),^†^ worked in health care occupations (23; 0.6%), or did not answer questions on hazard control use (22; 0.5%) were successively excluded to identify 742 (18%) non–health care, nonremote workers who did not use PPE at work before the COVID-19 pandemic. All further analyses were conducted using survey weights. This activity was reviewed by CDC and was conducted consistent with applicable federal law and CDC policy.^§^

Required and voluntary occupational use of hazard controls^¶^ and reasons for nonuse were described using percentages and 95% CIs as “Yes — my employer required it” (required use), “Yes — it was not required, but I used it” (voluntary use), “No — my employer did not allow it,” “No — I could not get any,” and “No — I did not think it was needed.” Voluntary use was then described among the subset of persons who were neither required to nor prohibited from using hazard controls (540); a survey-weighted regression model estimated the association between employer provision of hazard controls** and voluntary use as a risk difference. Models were adjusted for occupational group and proximity to others at work.^††^Respondents were classified as lower-income if the lower bound of the reported categorical household income was <250% of the 2019 national poverty threshold (*6*) based on reported household size and number of children. Stratum-specific aRDs were estimated to assess effect modification by income. All analyses were conducted using R software (version 4.0.2; The R Foundation).

Approximately one half (45.6%; 95% CI = 41.0%–50.3%) of non–health care workers reported use of occupational hazard controls (Figure). Most users of hazard controls (55.5%; 95% CI = 48.8%–62.2%) were required to do so by employers, and 44.5% (95% CI = 37.8%–51.2%) reported voluntary use. Among workers not using hazard controls, 8.1% (95% CI = 4.3%–11.8%) were prohibited from using them, 14.8% (95% CI = 9.9%–19.6%) could not obtain them, and 77.2% (95% CI = 71.5%–82.9%) did not believe they were needed. Overall, lower-income workers were more likely than were higher-income workers to be prohibited from using hazard controls (6.8%; 95% CI = 2.7%–10.9% versus 2.5%; 95% CI = 0.7%–4.3%) or to be unable to obtain them (12.6%; 95% CI = 6.9%–18.2% versus 4.5%; 95% CI = 2.5%–6.5%). Higher-income workers were more likely to report required use (27.7%; 95% CI = 22.4%–32.9%) and to use hazard controls overall (48.9%; 95% CI = 43.3%–54.4%) than were lower-income workers (22.3%; 95% CI = 15.5%–29.1% and 41.5%; 95% CI = 33.6%–49.3%, respectively).

**FIGURE:**
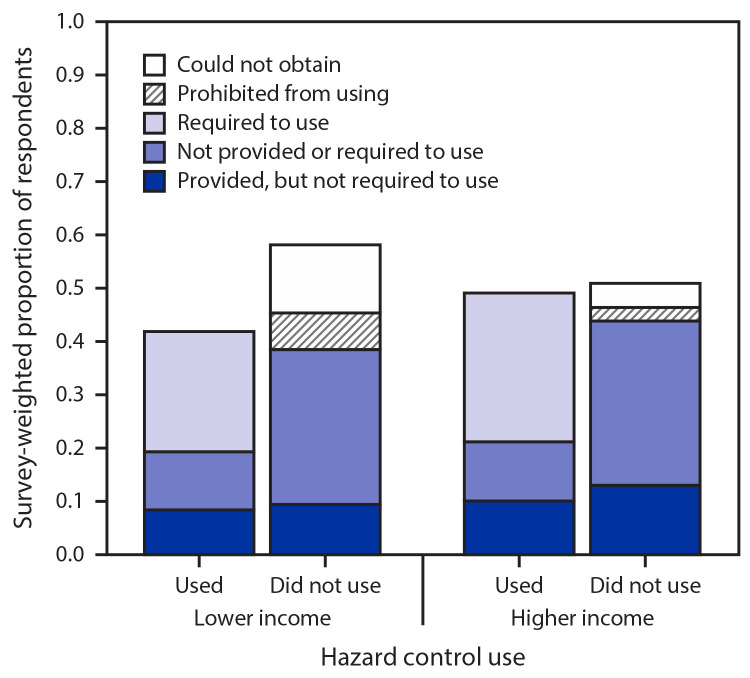
Reported occupational hazard control use for prevention of COVID-19 among survey respondents who reported primarily working outside the home in non–health care occupations after March 1, 2020, by household income* and workplace hazard control policies — 2020 SummerStyles, United States, June 2020 * Lower income: lower bound of the reported categorical household income <250% of the 2019 national poverty threshold based on household size and number of children; higher income: lower bound met or exceeded this threshold.

Among 540 workers for whom use of hazard controls was voluntary, 28.9% used them (Table 1). Some workers who were not required to use hazard controls were provided with them (29.7%; 95% CI = 24.8%–34.5%), and unadjusted voluntary use among workers provided with hazard controls (44.9%;) was twice that among those for whom hazard controls were not provided (22.4%;). After adjusting for occupational group and proximity to others at work, voluntary use was approximately double, or 22.3, absolute percentage points higher, among workers who were provided with hazard controls than among those who were not (Table 2). This effect was nonsignificantly larger among lower-income workers (aRD = 31.0%) than among higher-income workers (aRD = 16.3%).

**TABLE 1:** Survey-weighted proportions of occupational use of hazard controls among non–health care worker survey respondents who reported primarily working outside the home in settings where hazard control use was voluntary after March 1, 2020, by worker characteristics (N = 540) — 2020 SummerStyles, United States, June 2020

Characteristic (no. with available information if <540)	Voluntary hazard control use	
	Unweighted no. of respondents*	Survey-weighted % (95% CI)
**Total**	**540**	**28.9 (24.2–33.7)**
**Employer provided hazard controls (531)**		
Yes	175	44.9 (35.6–54.2)
No	356	22.4 (17.0–27.8)
**Sex**		
Male	284	27.9 (21.4–34.3)
Female	256	30.1 (23.1–37.1)
**Race/Ethnicity**		
White, non-Hispanic	424	26.8 (21.9–31.7)
Black, non-Hispanic	32	31.8 (13.7–49.9)
Other, non-Hispanic	38	36.7 (13.7–59.8)
Hispanic	46	34.9 (17.8–52.0)
**Age group, yrs**		
18–29	65	16.5 (6.3–26.7)
30–44	154	34.9 (26.3–43.5)
45–59	182	27.6 (19.9–35.2)
≥60	139	43.2 (33.9–52.5)
**Occupational group (511)**		
Professional and technical	201	25.9 (19.2–32.5)
Farming and production	61	26.1 (11.7–40.5)
Sales and office/Administrative support	84	30.8 (18.9–42.8)
Service	53	26.9 (13.7–40.1)
Other	112	36.1 (24.6–47.7)
**Proximity to others at work (within 6 ft) (538)**		
Never	119	21.9 (11.7–32.1)
Some of the time	188	33.2 (25.5–41.0)
Approximately one half of the time	73	30.4 (17.5–43.3)
Most of the time	85	25.9 (15.3–36.5)
Always	73	34.6 (19.8–49.4)
**Household income^†^**		
Lower income	193	27.0 (19.0–35.1)
Higher income	347	30.4 (24.8–36.0)

**Abbreviation:** CI = confidence interval.

* Unweighted sample size might not sum to column total because of missing values.

^†^ Lower income: lower bound of the reported categorical household income <250% of the 2019 national poverty threshold based on household size and number of children; higher income: lower bound met or exceeded this threshold.

**TABLE 2:** Adjusted, survey-weighted occupational use of hazard controls among non–health care worker survey respondents who reported primarily working outside the home in settings where hazard control use was voluntary after March 1, 2020, by employer provision of hazard controls and household income — 2020 SummerStyles, United States, June 2020

Characteristics	Respondent hazard control use,* % (95% CI)		
	Total	Lower household income^†^	Higher household income^†^
**Did employer provide hazard controls?**			
Yes	45.2 (36.1–54.3)	49.3 (31.8–66.7)	42.7 (33.0–52.5)
No	23.0 (17.7–28.3)	18.3 (10.7–25.9)	26.5 (19.6–33.4)
Risk difference*	22.3 (11.8–32.7)	31.0 (12.3–49.6)	16.3 (4.3–28.3)

**Abbreviation:** CI = confidence interval.

* Adjusted for occupational group and proximity to others at work. Adjusted hazard control use was estimated as a predictive marginal mean.

^†^ Lower income: lower bound of the reported categorical household income <250% of the 2019 national poverty threshold based on household size and number of children; higher income: lower bound met or exceeded this threshold.

## Discussion

Hazard controls, including physical barriers, cloth face masks, and other forms of PPE are important safeguards against occupational transmission of COVID-19 when work cannot be performed remotely (*1*,*2*). However, the use of COVID-19–specific hazard controls in non–health care workplaces is poorly characterized. A March 2020 survey, conducted before CDC’s recommendation for public mask use, reported that 7% of U.S. hourly-wage service-sector workers were required to use masks and that 19% were provided masks by their employers (*7*). The current analysis identified employer requirement and provision of hazard controls in June 2020 among a broader sample of occupations and found that many workers were neither provided with them nor required to use them. Employer provision of hazard controls was associated with greater use among all workers, particularly among lower-income workers.

Employers are required to provide a workplace free from recognized hazards (*8*). When engineering and administrative controls cannot fully protect workers, OSHA mandates that employers identify and provide necessary PPE at no cost to workers (*8*). Adherence to this mandate as employers adjust to new hazards posed by COVID-19 is vital to minimizing occupational transmission. CDC also recommends that employers encourage mask use to reduce transmission in workplaces where PPE is not routinely deemed necessary (*1*). Employer requirement and provision of hazard controls that are not considered PPE, such as cloth face masks and physical barriers, are complements to, not substitutes for, other workplace policies and worker protections (*1*,*2*).

Failure to protect workers from COVID-19 might exacerbate existing health disparities, including those among lower-income populations (*3*). Workers with lower incomes are more likely than are those with higher incomes to have preexisting health conditions that might increase the risk for severe COVID-19–associated illness (*3*). In this survey population, lower-income workers were also more likely to be unable to obtain or be prohibited from using occupational hazard controls. Cost to employees might hinder hazard control use, especially use of disposable items requiring regular replacement. Workers with lower incomes might also experience more job insecurity (*4*); workers should not be subjected to negative repercussions for reporting hazards and using hazard controls (*9*). A small minority of respondents, including a comparatively higher proportion of lower-income workers, reported being prohibited from using hazard controls by their employers. Targeted study can help identify reasons for such prohibition. Use of specific hazard controls should not be prohibited unless it impedes worker safety; in such situations, safe alternatives should be identified.

The findings in this report are subject to at least six limitations. First, survey questions did not distinguish between types of hazard controls despite differing implications for COVID-19 transmission. Second, employer provision of hazard controls was queried dichotomously. Respondents might have been provided some, but not all, recommended hazard controls. Third, some covariates were missing, and 7.0% of eligible workers were excluded from regression analyses because values were missing for one or more of the following: occupation (5.4%), employer provision of hazard controls (1.7%), or proximity to others at work (0.4%). Fourth, variance might be underestimated because sample design variables were unavailable; all analyses applied provided survey weights, treating the sample as a single stratum. Fifth, responses among this cross-sectional, English-language, opt-in panel sample might not be representative of the experiences of the broader workforce over time. Finally, small survey numbers within the relevant population produced large CIs for many estimates.

This analysis highlights the value of employer-provided hazard controls for increasing voluntary workplace use, particularly among workers with lower incomes. Employers can help protect workers against COVID-19 by requiring and encouraging occupational hazard control use and providing recommended hazard controls, along with other COVID-19 workplace precautions.

**Summary**

**What is already known about this topic?**

Certain hazard controls, including physical barriers, masks, and other personal protective equipment are recommended to reduce workplace COVID-19 transmission, but use is poorly characterized.

**What is added by this report?**

In June 2020, fewer than one half of nonremote, non–health care workers reported use of hazard controls to prevent COVID-19, and slightly more than one half of these reported required use. Voluntary use was approximately double (22 percentage points higher) among workers whose employers provided hazard controls than among those whose employers did not. This association was stronger among lower-income workers.

**What are the implications for public health practice?**

Employers can help protect workers against COVID-19 by requiring and encouraging occupational hazard control use and providing recommended hazard controls.

## Footnotes

* Porter Novelli Styles survey respondents received 5,000 reward points, equivalent to approximately $5, for completing the survey, but were not required to answer any of the questions and could choose to exit the survey at any point. Data are weighted to match U.S. Current Population Survey proportions for sex, age, household income, race/ethnicity, household size, education, census region, metro status, and parental status of adolescents aged 12–17 years. http://styles.porternovelli.com/consumer-youthstyles/

† The survey provided examples of PPE used before the COVID-19 pandemic to respondents, including “eye protection, gloves, a hard hat, a face mask, or a respirator.”

§ 45 C.F.R. part 46; 21 C.F.R. part 56; 42 U.S.C. Sect. 241(d), 5 U.S.C. Sect. 552a, 44 U.S.C. Sect. 3501 et seq.

¶ Use of occupational hazard controls for COVID-19 prevention was determined by the survey question “Since March 1, 2020, even though it is not normally required, did you wear any personal protective equipment (PPE) or use other physical barriers at work to prevent exposure to the virus that causes COVID-19?”

** Employer provision of hazard controls was determined by the survey question “Did your employer provide you with the PPE that you used or, if you chose not to use PPE, offer any to you?”

†† Occupation was collapsed into broad groups for analysis: 1) professional and technical, including management; business and financial operations; computer and mathematical; architecture and engineering; life, physical, and social sciences; community and social services; lawyer/judge; teacher, except college and university; teacher, college and university; other professional; business operations; financial operations or financial services; education, training, and library; and arts, design, entertainment, sports, and media; 2) farming and production, including farming, forestry, and fishing; construction and extraction; installation, maintenance, and repair; precision production; and transportation and material moving; 3) sales and office and administrative support, including sales representative; retail sales; other sales; office and administrative support; and sales; 4) service, including protective service; food preparation and serving; building and grounds cleaning and maintenance; and personal care and service; and 5) other, including armed services and other. Close proximity was classified as follows: required to perform job tasks within 6 feet of others always; most of the time; approximately one half of the time; some of the time; and never. Occupational group and close proximity to others at work were identified as potential confounders of the proposed association between employer provision of hazard controls and hazard control use after evaluating relationships among all covariates of interest using standard causal inference epidemiologic methods and were included as adjustment variables in regression models.
